# Development and validation of a prognosis prediction model based on 18 endoplasmic reticulum stress-related genes for patients with lung adenocarcinoma

**DOI:** 10.3389/fonc.2022.902353

**Published:** 2022-08-30

**Authors:** Long Shu, Shuang Liu, Yongguang Tao

**Affiliations:** ^1^ NHC Key Laboratory of Carcinogenesis (Central South University), Cancer Research Institute School of Basic Medicine, Central South University, Changsha, China; ^2^ Hunan Key Laboratory of Cancer Metabolism, Hunan Cancer Hospital and The Affiliated Cancer Hospital of Xiangya School of Medicine, Central South University, Changsha, China; ^3^ Department of Oncology, Institute of Medical Sciences, National Clinical Research Center for Geriatric Disorders, Xiangya Hospital, Central South University, Changsha, China; ^4^ Key Laboratory of Carcinogenesis and Cancer Invasion, Ministry of Education, Department of Pathology, Xiangya Hospital, School of Basic Medicine, Central South University, Changsha, China; ^5^ Hunan Key Laboratory of Early Diagnosis and Precision Therapy in Lung Cancer, Department of Thoracic Surgery, Second Xiangya Hospital, Central South University, Changsha, China

**Keywords:** prediction model, endoplasmic reticulum stress, lung adenocarcinoma, TCGA, GEO

## Abstract

**Background:**

Endoplasmic reticulum (ER) stress had a crucial impact on cell survival, proliferation, and metastasis in various cancers. However, the role of ER stress in lung adenocarcinoma remains unclear.

**Method:**

Gene expression and clinical data of lung adenocarcinoma (LUAD) samples were extracted from The Cancer Genome Atlas (TCGA) and three Gene Expression Omnibus (GEO) datasets. ER stress score (ERSS) was constructed based on hub genes selected from 799 ER stress-related genes by least absolute shrinkage and selection operator (LASSO) regression. A Cox regression model, integrating ERSS and the TNM stage, was developed to predict overall survival (OS) in TCGA cohort and was validated in GEO cohorts. Gene set enrichment analysis (GSEA), single-sample GSEA (ssGSEA), and gene mutation analyses were performed to further understand the molecular features of ERSS. The tumor immune infiltration was evaluated by ESTIMATE, CIBERSORT, and xCell algorithms. The receiver operating characteristic (ROC) curves were used to evaluate the predictive value of the risk model. *p*< 0.05 was considered statistically significant.

**Results:**

One hundred fifty-seven differentially expressed genes (DEGs) were identified between tumor and para-carcinoma tissues, and 45 of them significantly correlated with OS. Next, we identified 18 hub genes and constructed ERSS by LASSO regression. Multivariate analysis demonstrated that higher ERSS (*p*< 0.0001, hazard ratio (HR) = 3.8, 95%CI: 2.8–5.2) and TNM stage (*p*< 0.0001, HR = 1.55, 95%CI: 1.34–1.8) were independent predictors for worse OS. The prediction model integrating ERSS and TNM stage performed well in TCGA cohort (area under the curve (AUC) at five years = 0.748) and three GEO cohorts (AUC at 5 years = 0.658, 0.717, and 0.739). Pathway enrichment analysis showed that ERSS significantly correlated with unfolded protein response. Meanwhile, pathways associated with the cell cycle, growth, and metabolism were significantly enriched in the high ERSS group. Patients with *SMARCA4*, *TP53*, and *EGFR* mutations showed significantly higher ERSS (*p* = 4e−04, 0.0027, and 0.035, respectively). Tissues with high ERSS exhibited significantly higher infiltration of M1 macrophages, activated dendritic cells, and lower infiltration of CD8+ T cells and B cells, which indicate an activated tumor antigen-presenting but suppressive immune response status.

**Conclusion:**

We developed and validated an ER stress-related risk model that exhibited great predictive value for OS in patients with LUAD. Our work also expanded the understanding of the role of ER stress in LUAD.

## Introduction

Lung cancer is the most lethal malignant tumor worldwide (1) and contributes to the highest morbidity and mortality in China (2). Lung cancer consists of small cell carcinoma, adenocarcinoma, squamous cell carcinoma, and large cell carcinoma. Lung adenocarcinoma (LUAD) accounts for more than 40% of lung cancer, the most numerous histological type of lung cancer (3).

Under the condition of proteostasis in normal cells, the sensors of endoplasmic reticulum (ER) stress, including activating transcription factors 6 (*ATF6*), inositol-requiring enzyme 1α (*IRE1α*), and PRKR-like ER kinase (*PERK*), are in an inactivated state, while in the tumor microenvironment, multiple factors, such as hypoxia (4), abnormal nutrient supply (5), intracellular accumulation of reactive oxygen species (ROS) (6), and low pH (7), can disturb protein folding in ER. Accumulation of misfolded protein breaks proteostasis, activates sensors, and consequently drives robust ER stress in cancer cells. The activation of sensors promotes unfolded protein response (UPR), which restores ER homeostasis and promotes cell adaptation to stress and survival (8). Interestingly, ER stress acts as an oncogenic factor only when it is moderate, while extreme UPR caused by uncontrolled ER stress will induce cell death (9).

The role of ER stress in LUAD remains controversial. A study reported that ER stress was upregulated by the overexpression of *POU4F3* and therefore inhibits tumor progression in LUAD (10). ROS-mediated ER stress suppresses tumors in lung cancer cells (11). ER stress pathway can be upregulated by neutrophil arginase-1, released from activated human neutrophils or dead cells, and induces apoptosis of cancer cells (12). Besides, ER stress is also reportedly involved in cisplatin resistance in lung cells (13). In contrast, Yamashita et al. demonstrated that ER stress promotes epithelial–mesenchymal transition (EMT) and cell invasion in LUAD (14). Collectively, these findings indicate that ER stress might be a promising therapeutic target in LUAD, and comprehensive exploration of the relationship between ERSS stress and LUAD is necessary.

## Methods

### Datasets and data collection

Gene expression and clinical data of LUAD patients from The Cancer Genome Atlas (TCGA) database were obtained *via* the UCSC Xena repository (https://xenabrowser.net/) (15). The GSE30219, GSE31210, and GSE720924 datasets were procured from the Gene Expression Omnibus (GEO) database (http://www.ncbi.nlm.nih.gov/geo/). **Table 1** shows the clinical characteristics of patients from four cohorts. ER stress-related genes were downloaded from GeneCards websites (https://www.genecards.org/), which provides comprehensive information on all annotated human genes.

**Table 1:** Clinical characteristics of LUAD patients from TCGA and GEO databases.

	TCGA (n, %)	GSE30219 (n, %)	GSE31210 (n, %)	GSE72094 (n, %)
Total number	517 (100)	73 (100)	204 (100)	389 (100)
Age (years)				
<65	221 (42.7)	51 (69.9)	145 (71.1)	105 (27.0)
≥65	277 (53.6)	22 (30.1)	59 (28.9)	284 (73.0)
Unknown	19 (3.7)	0 (0)	0 (0)	0 (0)
Gender				
Male	240 (46.4)	56 (76.7)	95 (46.6)	172 (44.2)
Female	277 (53.6)	17 (33.3)	109 (53.4)	217 (55.8)
Smoking history				
Yes	427 (82.6)	–	105 (51.5)	297 (76.3)
No	76 (14.7)	–	99 (48.5)	29 (7.5)
Unknown	14 (2.7)	–	0 (0)	63 (16.2)
TNM stage				
I	277 (53.6)	69 (94.5)	162 (79.4)	252 (64.8)
II	122 (23.6)	3 (4.1)	42 (20.6)	65 (16.7)
III	84 (16.2)	1 (1.4)	0 (0)	57 (14.7)
IV	26 (5.0)	0 (0)	0 (0)	15 (3.8)
Unknown	8 (1.6)	0 (0)	0 (0)	0 (0)

LUAD, lung adenocarcinoma; TCGA, The Cancer Genome Atlas; GEO, Gene Expression Omnibus.

### Construction and evaluation of a prediction model

We developed a prediction model in TCGA cohort and externally validated the model in GEO cohorts. Differentially expressed genes (DEGs) between tumor and para-carcinoma tissues were identified by the limma R package in TCGA cohort. Univariate Cox regression analyses were conducted by survival R package to select overall survival (OS) related genes. Intersecting genes were identified and visualized by the VennDiagram R package. The intersecting genes were further analyzed by least absolute shrinkage and selection operator (LASSO) regression to seek OS-related genes further, using the glmnet R package. Expression heatmap of 18 hub DEGs was realized by ComplexHeatmap R package. The ER stress score (ERSS) was computed as follows:


$$ERSS=\sum_{k=1}^{n}{(}_{k}^{n})Coef_{k}\;*\;Exp_{k}$$

Exp_k_ is the expression value of the ERSS genes in the equation and Coef_k_ is the coefficient of each gene calculated by LASSO regression. The Kaplan–Meier survival plot and the Cox proportional-hazards regression were performed by the survival R package to clarify the predictive value of ERSS for OS. Our prediction model was constructed based on ERSS and Tumor-Node-Metastasis (TNM) stage using Cox proportional-hazards regression, as follows:


$$\text{Prognostic}\;index=Coef_{ERSS}\;*\;ERSS+Coef_{stage}\;*\;TNM\;stage$$

In this equation, Coef_ERSS_ means the coefficient of ERSS. Coef_stage_ is the coefficient of the TNM stage, including 1, 2, 3, and 4 (representing stages I, II, III, and IV, respectively). Nomogram was created by the rms R package to visualize the prediction model. Time-dependent receiver operating characteristic (ROC) analysis was performed by timeROC and survival R package to compare the predictive value of ERSS alone, TNM stage alone, and prediction model. For external validation, univariate survival, multivariate survival, and time-dependent ROC analyses were used to test the model’s performance in three GEO cohorts.

### Clinical and molecular feature analyses of endoplasmic reticulum stress score

To further explore the biological significance of ERSS, we analyzed the relationship between clinical, molecular, genetic, and immunological features and ERSS in patients with LUAD from TCGA cohort. The correlations between ERSS and clinical characteristics were analyzed in the ggpubr R package. Univariate Cox regression analyses were performed to calculate the hazard ratio (HR) and *p*-value of ERSS in different subgroups of patients. The results of subgroup survival analyses were visualized *via* forest plot by the forestplot R package. Patients were divided into ERSS high and low groups according to their median value for functional enrichment analysis. Then pathways of the hallmark, Gene Ontology (GO), and Kyoto Encyclopedia of Genes and Genomes (KEGG) were analyzed by the GSVA R package. The randomForest R package provided a random forest algorithm to screen gene mutations most related to ERSS. Estimation of STromal and Immune cells in MAlignant Tumours using Expression data (ESTIMATE) (16), cell type identification by estimating relative subsets of RNA transcripts (CIBERSORT) (17), and xCell (18) algorithms were performed to evaluate tumor infiltration of the immune cell by estimate, CIBERSORT, and xCell R packages, respectively.

### Statistical analyses

All statistical analyses and plots were accomplished in R software (4.1.0). DEGs were defined as *p*< 0.05 and fold change >2. The log-rank t-test was used to compare two survival curves in the Kaplan–Meier plot. The Wilcoxon test was applied to compare the statistical differences between the two groups with continuity values. The Kruskal–Wallis H test was employed to compare multiple groups with continuity values. *p*< 0.05 was considered statistically significant.

## Results

### Development of endoplasmic reticulum stress score based on 18 endoplasmic reticulum stress-associated genes

**Figure 1:**
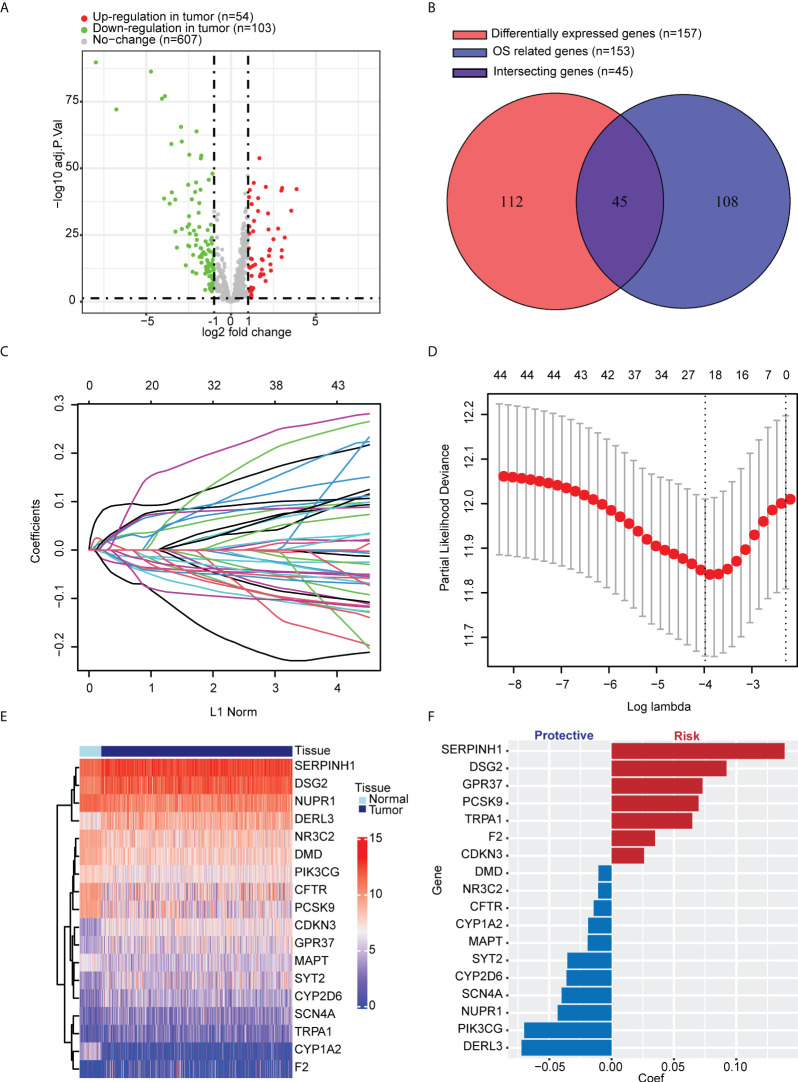
Identification of key ER stress-related genes correlated with tumorigenesis and survival in LUAD patients from TCGA cohort. **(A)** Among 764 ER stress-associated genes, 157 were DEGs between tumor and normal lung tissues. **(B)** One hundred fifty-three genes significantly correlated with OS in univariate Cox regression, 45 of which were DEGs. **(C, D)** LASSO regression and cross-validation identified 18 hub genes that correlated with OS. **(E)** Heatmap shows the expression of 18 hub genes. **(F)** Different hub genes had different coefficients. Among the 18 hub genes, seven correlated with a worse prognosis, and 11 correlated with a better prognosis. ER, endoplasmic reticulum; LUAD, lung adenocarcinoma; TCGA, The Cancer Genome Atlas; DEGs, differentially expressed genes; OS, overall survival; LASSO, least absolute shrinkage and selection operator.

We selected 799 ER stress-associated genes with a relevance score of >7 from the GeneCards database. Seven hundred sixty-four genes were detected in tumor tissue from LUAD patients in TCGA cohort (**Table S1**). One hundred fifty-seven DEGs were identified, comparing tumor and para-carcinoma tissues (**Figure 1**). In univariate Cox regression, 153 genes significantly correlated with OS. Forty-five intersecting genes were identified between DEGs and OS-related genes (**Figure 1**). Based on these intersecting genes, we performed LASSO regression and identified 18 OS-related hub genes (**Figures 1D**). The heatmap shows the relative gene expression of the 18 hub genes in tumor and para-carcinoma tissues (**Figure 1**). Integrating these 18 genes, we developed ERSS, which included 11 protective factors (*DMD*, *NR3C2*, *CFTR*, *CYP1A2*, *MAPT*, *SYT2*, *CYP2D6*, *SCN4A*, *NUPR1*, *PIK3CG*, and *DERL3*) and seven risk factors (*SERPINH1*, *DSG2*, *GPR37*, *PCSK9*, *TRPA1*, *F2*, and *CDKN3*) for LUAD survival (**Figure 1**). The equation of ERSS was as follows: ERSS = 0.13882291 * *SERPINH1* + 0.09216343 * *DSG2* + 0.07294183 * *GPR37* + 0.06978185 * *PCSK9* + 0.06473978 * *TRPA1* + 0.03490076 * *F2* + 0.02606376 * *CDKN3* − 0.01049731 * *DMD* − 0.01071941 * *NR3C2* − 0.01443196 * *CFTR* − 0.01872414 * *CYP1A2* − 0.01915136 * *MAPT* − 0.03551274 * *SYT2* − 0.03613574 * *CYP2D6* − 0.0400695 * *SCN4A* − 0.0432351 * *NUPR1* − 0.07008271 * *PIK3CG* − 0.0722234 * *DERL3*.

### Construction and evaluation of a prediction model by integrating endoplasmic reticulum stress score and TNM stage in patients with lung adenocarcinoma from The Cancer Genome Atlas cohort

**Figure 2:**
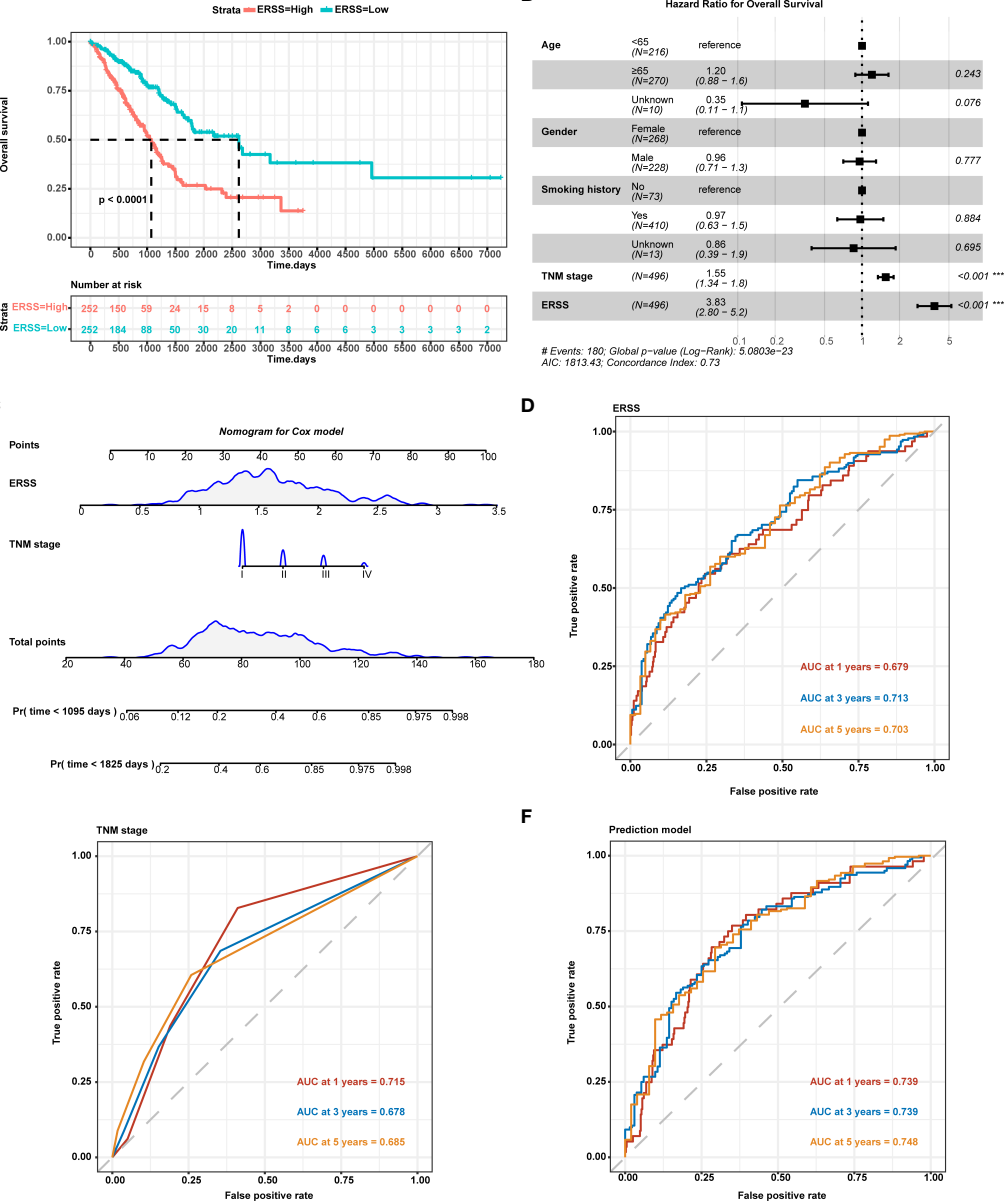
Constructing ERSS to predict OS in LUAD patients from TCGA cohort. **(A)** Patients with high ERSS showed significantly shorter OS (*p*< 0.0001). **(B)** Cox regression analysis showed that higher TNM stage (*p*< 0.001, HR = 1.55, 95%CI: 1.34–1.8) and higher ERSS (*p*< 0.001, HR = 3.83, 95%CI: 2.8–5.2) were independent predictors for shorter OS. **(C)** We constructed a prediction model consisting of the TNM stage and ERSS by Cox regression. Nomogram was used to visualize the Cox model. **(D)** AUC at 1, 3, and 5 years of ERSS for OS. **(E)** AUC at 1, 3, and 5 years of TNM stage for OS. **(F)** AUC at 1, 3, and 5 years of the prediction model for OS. ERSS, endoplasmic reticulum stress score; LUAD, lung adenocarcinoma; TCGA, The Cancer Genome Atlas; OS, overall survival; ROC, receiver operating characteristic; AUC, the area under curve. ***p <0.0001.


Compared with the low ERSS group, the high ERSS group showed significantly shorter OS (*p*< 0.0001; **Figure 2**). Cox proportional-hazards regression demonstrated that higher ERSS (*p*< 0.0001; HR = 3.8, 95%CI: 2.8–5.2) and TNM stage (*p*< 0.0001; HR = 1.55, 95%CI: 1.34–1.8) were independent predictors for worse OS (**Figure 2**). Integrating ERSS and TNM stage, we constructed a prediction model and visualized the model by nomogram (**Figure 2**). The equation of the prediction model was as follows:

Prognostic index=1.563 * TNM Stage + 3.709 * ERSS

ROC curve analysis showed that ERSS alone had a great predictive value for OS (area under the curve (AUC) at 5 years = 0.703; **Figure 2**), while the TNM stage showed a lower predictive value (AUC at 5 years = 0.685; **Figure 2**). Interestingly, our prediction model showed a significantly higher predictive value for OS (AUC at 5 years = 0.748) than for ERSS or TNM stage alone (**Figure 2**).

### External validation of the prediction model in Gene Expression Omnibus datasets

**Figure 3:**
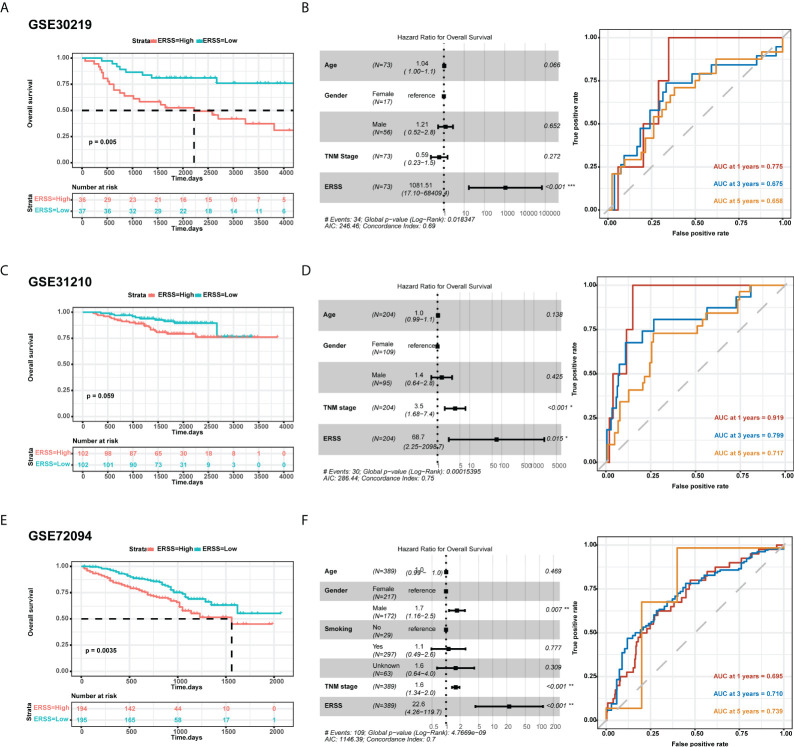
External validation of ERSS in GEO cohorts. **(A, B)** Validation in GSE30219 cohort showed that high ERSS significantly correlated with shorter OS (*p* = 0.005) and independently predicted shorter OS (*p*< 0.001). **(C)** The AUC values of ERSS for 1-, 3-, and 5-year OS were 0.775, 0.675, and 0.658, respectively. **(D)** In the GSE31210 cohort, patients with high ERSS showed shorter OS (*p* = 0.059). **(E)** Higher ERSS was an independent predictor for worse OS (*p* = 0.015). **(F)** The AUC values of ERSS for 1, 3, and 5 years OS were 0.919, 0.799, and 0.717, respectively. **(G, H)** Similarly, univariate and multivariate analyses showed that high ERSS predicts worse OS in the GSE72094 cohort. **(F)** The AUC values of ERSS for 1, 3, and 5 years OS were 0.695, 0.710, and 0.739, respectively. ERSS, endoplasmic reticulum stress score; OS, overall survival; AUC, the area under curve. *p <0.05, **p <0.01, ***p <0.0001.

To further validate the predictive value of the prediction model, we used three cohorts from GEO datasets for external validation. In the GSE30219 cohort, 73 patients were diagnosed with LUAD, more than 90% of whom were stage I. Patients with high ERSS had significantly shorter OS in univariate analysis and multivariate analysis (*p* = 0.005 and *p*< 0.001, respectively; **Figures 3B**). AUC values at 1, 3, and 5 years of the prediction model were 0.775, 0.675, and 0.658, respectively (**Figure 3**). Interestingly, the TNM stage showed no significant contribution to OS in multivariate analysis. In GSE31210 datasets, 204 patients were LUAD. Although the statistical difference was insignificant in the Kaplan–Meier survival analysis (*p* = 0.059; **Figure 3**), high ERSS was a substantial risk factor for worse OS in Cox regression analysis (*p* = 0.015; **Figure 3**). The prediction model had an excellent predictive value for predicting 1-year survival (AUC = 0.919; **Figure 3**) in this cohort. In another cohort, GSE72094, which had the most significant number of LUAD patients (n = 389), ERSS still played as an independent predictor (*p* = 0.0035 for univariate analysis and *p*< 0.001 for multivariate analysis; **Figures 3H**). Our prediction model also performed well in this cohort (AUC at 1, 3, and 5 years = 0.695, 0.710, and 0.739, respectively; **Figure 3**).

### The correlation between clinical characteristics and endoplasmic reticulum stress score

**Figure 4:**
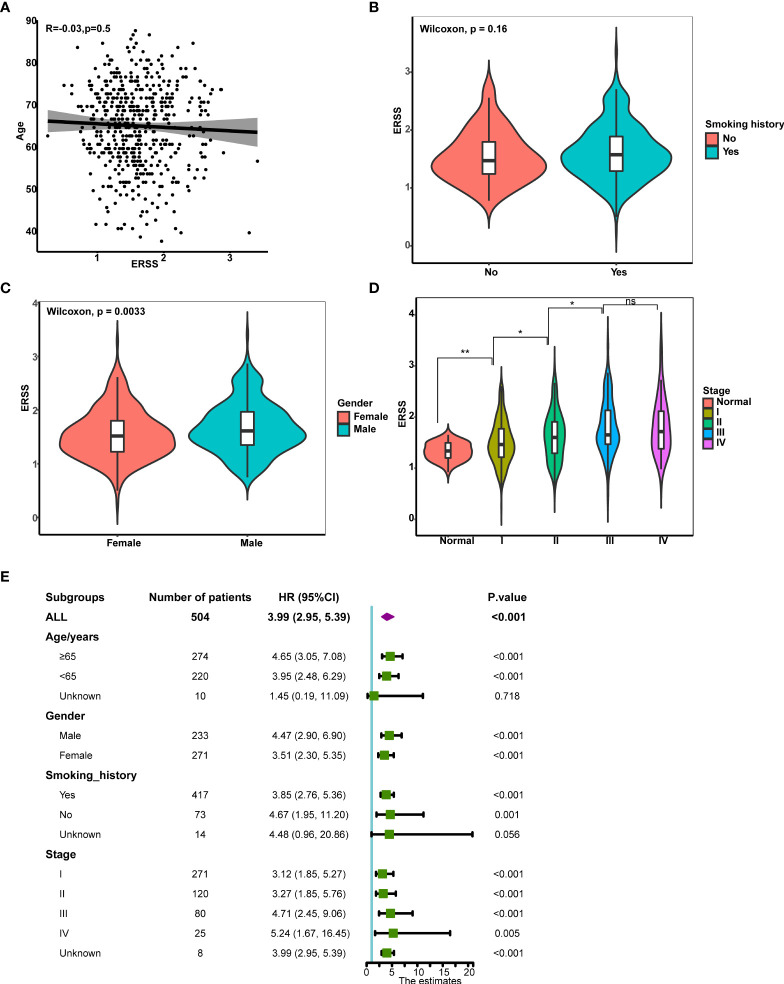
The correlation between clinical features and ERSS in TCGA cohort. **(A)** ERSS had no correlation with age. **(B)** Men had higher ERSS compared with women. **(C)** Smoking history had no association with ERSS. **(D)** Patients with advanced stage showed significantly higher ERSS than patients with early stage. **(E)** High ERSS significantly correlated with worse OS in different subgroups. ERSS, endoplasmic reticulum stress score; TCGA, The Cancer Genome Atlas; OS, overall survival. *p <0.05, **p <0.01, ns means *p* >0.05.

Next, we analyzed the correlation between clinical characteristics with ERSS and the predictive value of ERSS in different subgroups in TCGA cohort. ERSS had no correlation with age or smoking history (*p* = 0.5 and 0.16, respectively; **Figures 4B**). Women had significantly lower ERSS as compared with men (*p* = 0.0033; **Figure 4**). Besides, higher ERSS also correlated with the higher TNM stage (**Figure 4**). For subgroup analysis, higher ERSS still predicts worse OS in most of the subgroups (**Figure 4**). It should be noted that ERSS had a significant association with OS in all stages, whether in the early stage or advanced stage.

### The molecular features of endoplasmic reticulum stress score

**Figure 5:**
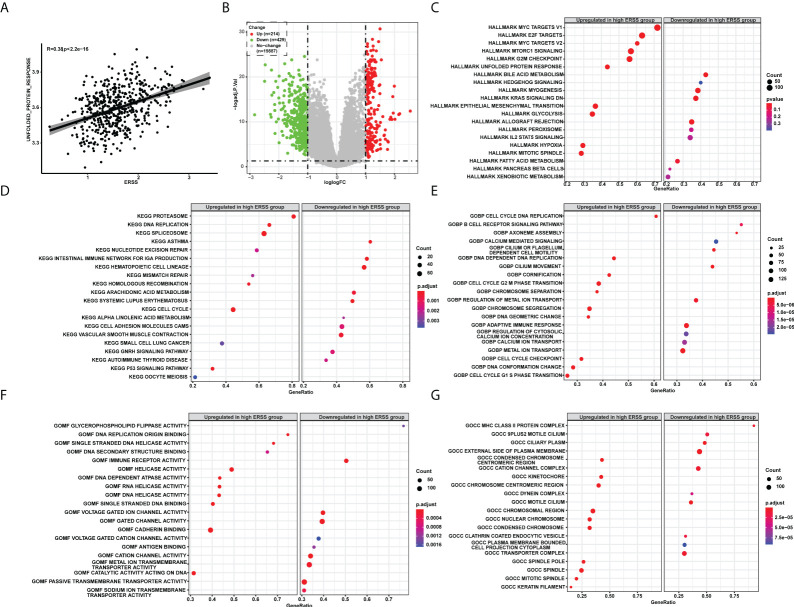
The pathway enrichment analysis explored molecular features of ERSS in TCGA cohort. **(A)** Single-sample GSEA showed that ERSS significantly and positively correlated with unfolded protein response pathway. **(B)** Differentially expressed genes were analyzed between patients with high ERSS and low ERSS. **(C)** The hallmark pathways significantly activated or suppressed in high ERSS group. The top 20 significant KEGG **(D)**, GO BP **(E)**, MF **(F)**, and CC **(G)** terms enriched in high ERSS group. ERSS, endoplasmic reticulum stress score; GSEA, gene set enrichment analysis; KEGG, Kyoto Encyclopedia of Genes and Genomes; GO, Gene Ontology; BP, biological process; MF, molecular function; CC, cell component.

We next sought to determine the molecular features of ERSS. Unfolded protein response pathway is closely related to the degree of ER stress. Thus, we first calculated the UPR by single single-sample gene set enrichment analysis (ssGSEA) and analyzed the correlation between UPR and ERSS, and we found that ERSS positively correlated with UPR (R = 0.38, *p*< 2.2e−16; **Figure 5**). Compared with the low ERSS group, 643 DEGs were found in the high ERSS group (**Figure 5**). Hallmark pathway enrichment showed that the top five gene ratio pathways were Myc targets V1, E2F targets, Myc targets V2, mTORC1 signaling, and MARK G2M Checkpoint (**Figure 5**). The top five significant KEGG terms enriched were Proteasome, DNA replication, spliceosome, asthma, and nucleotide excision repair (**Figure 5**). The top five important GO BP terms were cell cycle DNA replication, B-cell receptor signaling pathway, axoneme assembly, calcium-mediated signaling, and cilium or flagellum-dependent cell motility (**Figure 5**). The top five significant GO MF terms were glycerophospholipid flippase activity, DNA replication origin binding, single-stranded DNA helicase activity, DNA secondary structure binding, and immune receptor activity (**Figure 5**). The top five significant GO CC terms were MHC class II protein complex, 9PLUS2 motile cilium, ciliary plasm, external side of the plasma membrane, and condensed chromosome centromeric region (**Figure 5**).

### Endoplasmic reticulum stress score was associated with driver gene mutations

**Figure 6:**
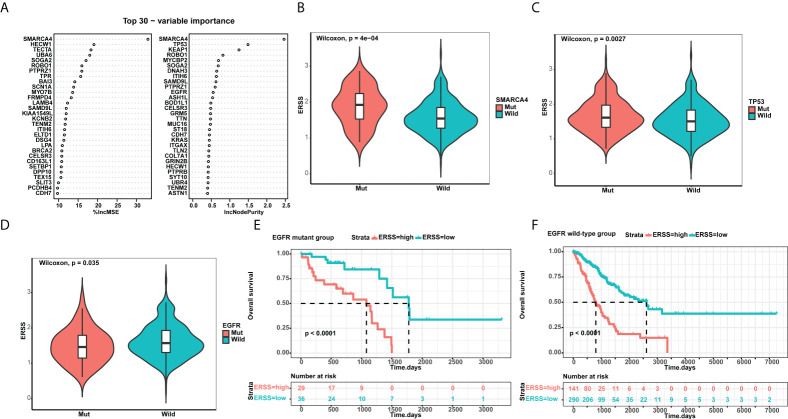
ERSS correlated with driver gene mutations. **(A)** Random forest selected the top 30 important mutant genes that associated with ERSS. **(B, C)** Patients with *SMARCA4* and *TP53* mutations showed significantly higher ERSS (*p* = 4e−04 and 0.0027, respectively). **(D)** Patients with *EGFR* mutation showed lower ERSS (*p* = 0.035). **(E, F)** Patients with high ERSS had significantly longer OS neither in *EGFR* mutant nor in wild-type group (both *p*< 0.0001). ERSS, endoplasmic reticulum stress score; TCGA, The Cancer Genome Atlas; OS, overall survival.

Using a random forest algorithm, we selected gene mutations closely correlated with ERSS and listed genes with top 30 importance (**Figure 6**). *SMARCA4* mutation had the highest importance, and patients with *SMARCA4* mutation showed significantly higher ERSS (*p* = 4e−04; **Figure 6**). Meanwhile, we found driver gene mutations were also associated with ERSS. *TP53* mutation group had substantially higher ERSS (*p* = 0.0027; **Figure 6**). In contrast, patients with *EGFR* mutation had lower ERSS (*p* = 0.035; **Figure 6**), which aroused our interest in exploring the prognostic significance of ERSS in patients with different *EGFR* mutation statuses. The results showed that high ERSS predicted worse OS, whether in patients with *EGFR* mutation or wild-type (both *p*< 0.0001; **Figures 6F**).

### Endoplasmic reticulum stress score was associated with tumor infiltration of lymphocytes

**Figure 7:**
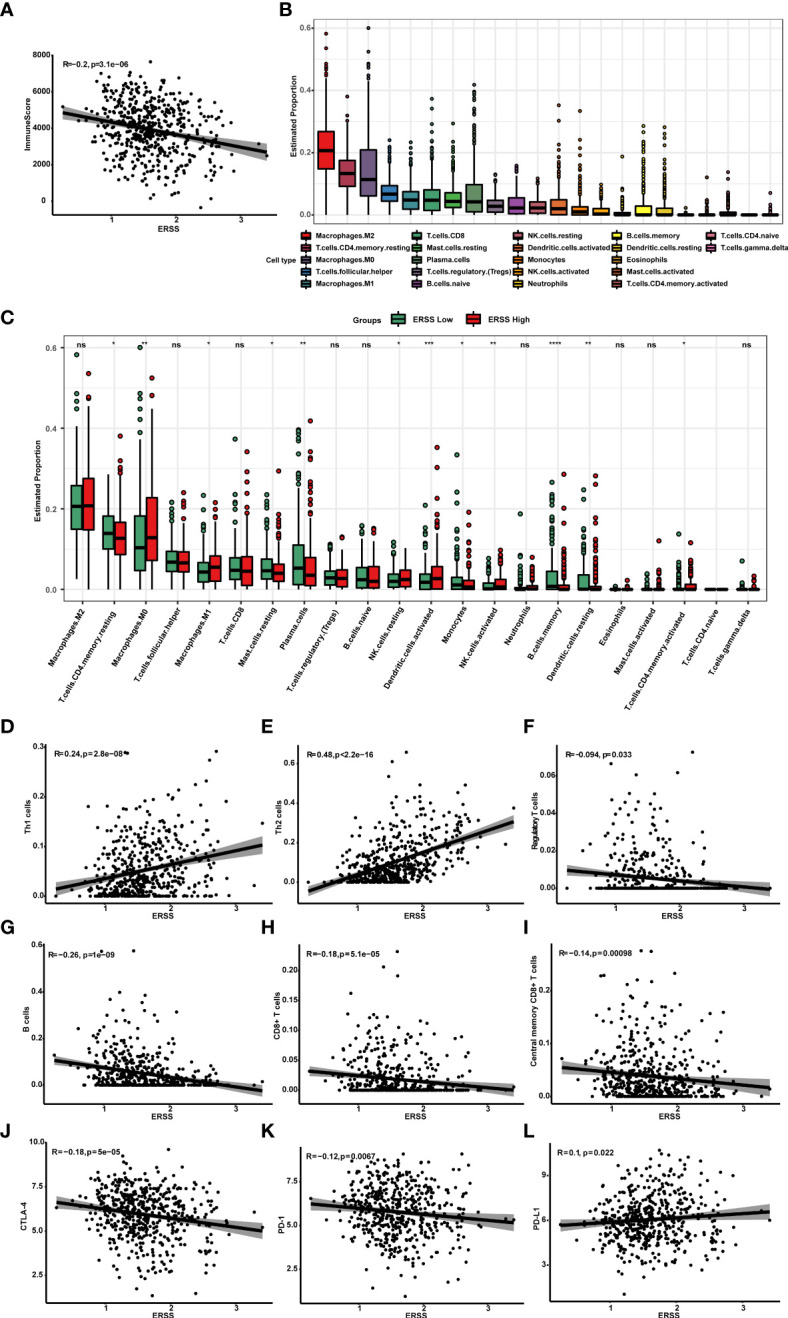
High ERSS correlated with a suppressive tumor immune microenvironment. **(A)** ESTIMATE algorithms showed that ERSS negatively correlated with immune score (*p* = 3.1e−06, R = −0.2), suggesting that higher ERSS was accomplished with less immune cell infiltration. **(B)** Twenty-two types of immune cells were evaluated by CIBERSORT algorithms. M2 macrophages accounted for the highest proportion of all immune cells, followed by resting CD4+ memory T cells. **(C)** The level of infiltrating immune cells was well compared by a multi-group box diagram between high and low ERSS groups (median value of ERSS was used to distinguish high and low groups). Compared with the low ERSS groups, the high ERSS group had significantly higher infiltration of M0 macrophages, M1 macrophages, resting NK cells, activated dendritic cells, and activated CD4+ memory T cells and lower infiltration of resting CD4+ memory T cells, resting mast cells, plasma cells, monocytes, memory B cells, and resting dendritic cells. **(D–F)** We performed xCell algorithms to further clarify the changes in different subtypes of CD4+T cells. Th1 cells (*p* = 2.8e−08, R = 0.24) and Th2 cells (*p*< 2.2e-16, R = 0.48) were positively with ERSS. Regulatory T cells were negatively associated with ERSS (*p* = 0.033, R = −0.094). **(G–I)** Meanwhile, xCell algorithms showed that ERSS negatively correlated with infiltration of B cells (*p* = 1e−09, R = −0.26), CD8+ T cells (*p* = 5.1e−05, R = −0.18), and central memory CD8+ T cells (*p* = 0.00098, R = −0.14). **(J–L)** Higher ERSS was significantly associated with lower expression of CTLA-4 (*p* = 5e−05, R = −0.18) and PD-1 (*p* = 0.0067, R = −0.12) and higher expression of PD-L1 (*p* = 0.022, R = 0.1). ERSS, endoplasmic reticulum stress score; TCGA, The Cancer Genome Atlas; LUAD, lung adenocarcinoma. *p <0.05, **p <0.01, ***p <0.0001, and ns means *p* >0.05.

To understand the association between ERSS and tumor immune microenvironment, we performed ESTIMATE, CIBERSORT, and xCell algorithms in patients with lung adenocarcinoma from TCGA database and analyzed the relationship between ERSS and immune checkpoints. The immune score was calculated by ESTIMATE algorithms and represents the abundance of tumor infiltration of immune cell negatively correlated with ERSS (*p* = 3.1e−06, R = −0.2; **Figure 7**), which suggested that higher ERSS was accomplished with less immune cell infiltration. CIBERSORT algorithms showed that M2 macrophages accounted for the highest proportion of all immune cells, followed by resting CD4+ memory T cells (**Figure 7**). Compared with the low ERSS groups, the high ERSS group had significantly higher infiltration of antigen-presenting cells, such as M1 macrophages and activated dendritic cells, but lower memory B cells (**Figure 7**). The xCell algorithms showed that Th1 cells (*p* = 2.8e−08, R = 0.24) and Th2 cells (*p*< 2.2e−16, R = 0.48) were positively associated and regulatory T cells (*p* = 0.033, R = −0.094) were negatively associated with ERSS (**Figures 7–F**). Meanwhile, xCell algorithms showed that ERSS negatively correlated with infiltration of B cells (*p* = 1e−09, R = −0.26), CD8+ T cells (*p* = 5.1e−05, R = −0.18), and central memory CD8+ T cells (*p* = 0.00098, R = −0.14) (**Figures 7–I**). Gene expression association analyses suggested that higher ERSS was significantly associated with lower expression of CTLA-4 (*p* = 5e−05, R = −0.18) and PD-1 (*p* = 0.0067, R = −0.12) and higher expression of PD-L1 (*p* = 0.022, R = 0.1) (**Figures 7–L**).

## Discussion

Machine learning has many advantages in analyzing datasets with large samples and features compared with traditional biostatistical methods, which makes it deployable to build prediction models for survival and treatment efficacy in cancer patients (19, 20). LASSO and Cox regression analyses are commonly employed as machine learning methods to develop risk models (21). In this study, we identified 18 hub genes among 799 ER stress-related genes in 563 patients with LUAD by LASSO regression. Cox regression analysis demonstrated that higher ERSS and TNM stage can independently predict worse OS. Integrating both, we constructed a simple and efficient prediction model. To promote the clinical application of the model, we also developed a nomogram only combing ERSS and TNM stages. Previous studies have reported risk models for LUAD (22–26). The following differences distinguish our study from other prediction models. First, external validation is one of the most important parts of model destruction. Some studies used one or two external cohorts (25, 26), while some only used internal validation (22–24). We used three independent external cohorts, and the model performed well in all cohorts. Second, our model consists of ER stress-related hub genes. Thus, the model also expanded our understanding of ER stress in LUAD.

Of the 18 hub genes, 11 were protective factors (*DMD*, *NR3C2*, *CFTR*, *CYP1A2*, *MAPT*, *SYT2*, *CYP2D6*, *SCN4A*, *NUPR1*, *PIK3CG*, and *DERL3*), and seven were risk factors (*SERPINH1*, *DSG2*, *GPR37*, *PCSK9*, *TRPA1*, *F2*, and *CDKN3*). *DMD*, the Duchenne muscular dystrophy gene, encodes dystrophin protein and is known for its role in the disease of the same name (27). Multiple studies reported that *DMD* suppresses tumor progression in human cancer (28). *NR3C2* (nuclear receptor subfamily 3, group C, member 2) is a transcription factor and encodes mineralocorticoid receptor protein, which inhibits cancer angiogenesis (29). A recent study demonstrated that *NR3C2* suppresses colon cancer progression by inhibiting the AKT/ERK pathway (30). *CFTR* (cystic fibrosis transmembrane conductance regulator), which belongs to the ATP-binding cassette transporter superfamily, regulates several fundamental cellular processes, including development and epithelial differentiation (31). Studies reported that *CFTR* acts as a tumor suppressor and is downregulated in lung cancer (32), and dysfunctional *CFTR* is associated with cancer progression (33). *CYP1A2* (cytochrome P450 1A2) and *CYP2D6* (cytochrome P450 2D6) both belong to the cytochrome P450 superfamily, which regulates the metabolism of commonly used drugs and is predominantly distributed in the liver (34). In hepatocellular carcinoma, *CYP1A2* inhibits cancer progression through antagonizing HGF/MET signaling (35). *CYP2D6* is necessary for the activation of tamoxifen, and higher expression of *CYP2D6* is associated with better survival in patients with breast cancer (36). *MAPT*, encoding microtubule-associated protein tau, plays an important role in nervous system disease (37). Low expression of *MAPT* has been linked to poor prognosis in prostate and clear cell renal cell cancer (38, 39). *NUPR1* (nuclear protein 1) reduces ER stress by interacting with eIF2α (40) and plays a tumor promoter role in lung cancer (41, 42). However, the influence of *NUPR1* on cancer behavior is still unclear (43). *PIK3CG* is a candidate suppressor for myeloid tumors (44). Silencing the *PIK3CG* inhibits the PI3K-Akt/PKB pathway, resulting in tumorigenesis and progression of colorectal cancer (45). However, recent studies demonstrated that *PIK3CG* promotes tumor progression in prostate cancer and breast cancer (46, 47). *DERL3* encodes a derlin-3 protein that belongs to the Derlin family and functions in the endoplasmic reticulum. During the UPR, *DERL3* is upregulated by *ATF6* and enhances the degradation of misfolded proteins (48). Several studies reported the role of *DERL3* as a tumor suppressor in colorectal (49), gastric (50), and lung cancers (51). *SERPINH1* (serpin peptidase inhibitor, clade H, member 1) is a kind of serine proteinase inhibitor. A pan-cancer analysis reported that *SERPINH1* strongly correlated with worse survival in various cancers (52). *DEG2* (desmoglein2) is a component of the desmosome-mediated intercellular adhesion complex. High expression of *DEG2* correlated with poor survival in patients with colon (53), cervical (54), and lung cancers (55). A recent study demonstrated that *DEG2* mediated hypoxia-derived tumor metastasis in breast cancer (56). *PCSK9* (proprotein convertase subtilisin/kexin type-9), a critical protein that regulates cholesterol metabolism, promotes the incidence and progression of several cancers (57). A recent study reported that *PCSK9* inhibits the expression of *MHC-I* on tumor cells and consequently decreases tumor infiltration of cytotoxic T cells. Meanwhile, inhibiting *PCSK9* effectively enhances PD-1 inhibitor therapy for cancers (58). *TRPA1* belongs to the transient receptor potential family and regulates the transportation of Ca(2+). *TRPA1* decreases ROS accumulation in cancer cells and thus promotes cell survival (59). *CDKN3* (cyclin-dependent kinase inhibitor 3) plays a vital role in cell cycle regulation. Deletion, mutation, and overexpression of *CDKN3* were associated with tumor progression in several cancers (60, 61). The role of *SYT2*, *SCN4A*, *GPR37*, and *F2* in cancers has not been well studied.

To expand our understanding of the role of ERSS in LUAD, we further exploited the molecular features of ERSS. Enrichment analysis showed that besides UPR, pathways related to cell cycle, growth, and metabolism were significantly enriched in the high ERSS group, suggesting that ER stress was closely correlated with tumorigenesis and progression. The status of gene mutation and tumor immune microenvironment (TIME) play crucial roles in the treatment strategies for patients with LUAD. This study found that *SMARCA4* mutation is the most relevant mutation to ERSS, and patients with *SMARCA4* mutation showed significantly higher ERSS. *SMARCA4*, which encodes a fundamental unit of SWI/SNF (switch/sucrose non-fermentable) chromatin remodeling complex, acts as a tumor suppressor but is frequently inactivated by mutations in non-small cell lung cancer (NSCLC) (62). In patients with metastatic NSCLC, *SMARCA4* mutation significantly correlated with shorter OS (63). Currently, no researchers have reported the relationship between *SMARCA4* mutation and ER stress. *EGFR* mutations account for nearly 50% of Asian patients with advanced NSCLC. Tyrosine kinase inhibitors (TKIs) that targeted *EGFR* mutation were the standard first-line treatment for these patients (64, 65). However, *EGFR*-targeted therapy usually develops drug resistance after 10–14 months (66). An *in vivo* study reported that ROS-mediated ERSS might affect the efficacy of EGFR inhibitors in *EGFR* wild-type cells (67). We found that patients with EGFR mutation had significantly higher ERSS, and ERSS was a powerful predictor for prognosis in patients with *EGFR* mutation, which indicates that ERSS might help overcome EGFR-TKI resistance by screening high-risk population in *EGFR* mutant patients. The immune phenotype analysis demonstrated that the high ERSS group had a distinct tumor immune microenvironment as compared with a low ERSS group. Tumors from patients with high ERSS had higher infiltration of antigen-presenting cells, such as M1 macrophages and activated dendritic cells. However, ERSS negatively correlated with the infiltration of CD8+ T cells and B cells and positively correlated with the expression of PD-L1. These results indicated that the antigen presentation in tumor tissue with higher ERSS might be more activated, but the anti-tumor function was more suppressive because of other regulatory factors, such as the increased expression of PD-L1. Considering the close correlation between ERSS and the immune status of TIME, it is worthwhile to further explore the role of ER stress in immune therapy.

This study has several limitations. First, we did not validate the predicted value of the 18 hub genes by experiments *in vitro* or *in vivo*. More functional experiments are necessary to specify the biological roles of every hub gene. Second, although the ERSS showed independent predictive value for OS in external cohorts from GEO datasets, further research remains necessary to confirm the performance of ERSS in expanded cohorts. In addition, our study indicated that ERSS correlated with the efficacy of targeted therapy and immune therapy; however, the correlation needs to be validated in other patients under treatment.

## Data availability statement

Publicly available datasets were analyzed in this study. Gene expression and clinical data of LUAD patients from the Cancer Genome Atlas (TCGA) database were obtained *via* the UCSC Xena repository (https://xenabrowser.net/) (15). The GSE30219, GSE31210, and GSE720924 datasets were procured from the Gene Expression Omnibus (GEO) database (http://www.ncbi.nlm.nih.gov/geo/).

## Author contributions

Contribution: (I) Conception and design: YT; (II) Collection and assembly of data: LS and SL; (III): Data analysis and interpretation: LS and SL; (IV) Manuscript writing: LS; (V) Final approval of manuscript: All authors.

## Funding

This work was supported by the National Natural Science Foundation of China [82072594, YT; 82073097, 81874139, SL], Natural Science Foundation of Hunan Province, and Hunan Provincial Key Area R&D Programs [2021SK2013, YT].

## Conflict of interest

The authors declare that the research was conducted in the absence of any commercial or financial relationships that could be construed as a potential conflict of interest.

## Publisher’s note

All claims expressed in this article are solely those of the authors and do not necessarily represent those of their affiliated organizations, or those of the publisher, the editors and the reviewers. Any product that may be evaluated in this article, or claim that may be made by its manufacturer, is not guaranteed or endorsed by the publisher.
