# Supplementary material for: Development and validation of a prognosis prediction model based on 18 endoplasmic reticulum stress-related genes for patients with lung adenocarcinoma
Source: Front Oncol. 2022 Aug 30;12:902353. doi: 10.3389/fonc.2022.902353 (PMC9469654; doi:10.3389/fonc.2022.902353)
Supplement: Supplementary file 1 [file Table_1.docx]

**Table S1. The 764 ER stress-related genes.**

| Gene Symbol | Relevance score |  | Gene Symbol | Relevance score |
| --- | --- | --- | --- | --- |
| ERN1 | 66.36483002 |  | STX18 | 10.03302574 |
| HSPA5 | 66.15071869 |  | ACTB | 10.01654434 |
| ATP2A2 | 57.77500534 |  | TMED10 | 10.00955868 |
| EIF2AK3 | 52.16033936 |  | HRC | 10.00540733 |
| XBP1 | 49.2240181 |  | TRPV4 | 9.985916138 |
| ATP2A1 | 48.24971008 |  | SP1 | 9.985847473 |
| ATP2A3 | 45.96712875 |  | CYBB | 9.983383179 |
| SERP1 | 45.88234711 |  | DNM1L | 9.961863518 |
| ERP29 | 45.39149475 |  | BCL2L11 | 9.912500381 |
| OS9 | 42.38906479 |  | GANAB | 9.87925148 |
| ERP44 | 42.22026825 |  | HLA-A | 9.864295006 |
| SERP2 | 42.09656525 |  | VKORC1 | 9.861377716 |
| HERPUD1 | 41.94785309 |  | RHOA | 9.856463432 |
| VCP | 41.30079269 |  | SEC31B | 9.849932671 |
| KDELR1 | 40.22213364 |  | TOR1AIP2 | 9.833639145 |
| ERAP1 | 40.18216705 |  | UBQLN2 | 9.827015877 |
| ATF6 | 39.76169586 |  | TIA1 | 9.821984291 |
| KDELR2 | 38.38109589 |  | ACTA1 | 9.814920425 |
| ERLEC1 | 38.24609375 |  | RYR3 | 9.78751564 |
| ERAP2 | 36.85629272 |  | CDKN1A | 9.761175156 |
| DDIT3 | 36.47018051 |  | PON2 | 9.758685112 |
| SEC16A | 35.62680435 |  | HSPD1 | 9.758406639 |
| ERN2 | 35.18235016 |  | THBS1 | 9.750688553 |
| KDELR3 | 35.07497787 |  | STX5 | 9.748083115 |
| CALR | 34.81806183 |  | SRC | 9.747422218 |
| ERP27 | 34.57245255 |  | NOTCH1 | 9.742071152 |
| TP53 | 34.55228043 |  | COMP | 9.708352089 |
| HSP90B1 | 33.92495728 |  | VCAM1 | 9.703249931 |
| RER1 | 32.9659729 |  | ELN | 9.688709259 |
| CANX | 32.54264069 |  | APEX1 | 9.687224388 |
| ATF4 | 32.45502472 |  | ZDHHC6 | 9.682829857 |
| CHERP | 32.1427269 |  | ICMT | 9.678264618 |
| RYR1 | 31.7507534 |  | TMCC1 | 9.654649734 |
| EIF2S1 | 30.50608063 |  | PCSK6 | 9.641375542 |
| RYR2 | 30.03034592 |  | TMX3 | 9.639509201 |
| SYVN1 | 29.64959526 |  | ALG13 | 9.639347076 |
| ERMP1 | 29.26678848 |  | DMPK | 9.6295681 |
| PDIA3 | 28.29377747 |  | MAPK13 | 9.62121582 |
| MAPK8 | 27.88607979 |  | SSR2 | 9.6027174 |
| CPT2 | 26.77774239 |  | MAN1A1 | 9.589347839 |
| SIL1 | 26.35389709 |  | KCNJ11 | 9.560453415 |
| DERL2 | 25.76849747 |  | SLC2A1 | 9.555160522 |
| **Table S1. The 764 ER stress-related genes.** | | | | |
| Gene Symbol | Relevance score |  | Gene Symbol | Relevance score |
| TRDN | 25.5766468 |  | CYP1A1 | 9.534471512 |
| NFE2L2 | 25.31622314 |  | KCNE2 | 9.527271271 |
| CASQ2 | 25.16840935 |  | BRSK2 | 9.518390656 |
| WFS1 | 25.11867523 |  | EP300 | 9.497811317 |
| DERL1 | 25.03804016 |  | MAOA | 9.481782913 |
| NFE2L1 | 24.9792881 |  | NGLY1 | 9.47981739 |
| DNAJC3 | 24.76097679 |  | PPARGC1A | 9.466560364 |
| DNAJC10 | 24.63835144 |  | SEC22B | 9.453193665 |
| LMAN1 | 24.59807014 |  | MAP2K6 | 9.438858986 |
| SOD1 | 24.34274292 |  | TMEM199 | 9.437177658 |
| P4HB | 23.87190628 |  | OSBP | 9.430151939 |
| HYOU1 | 23.84248734 |  | SRP68 | 9.426178932 |
| APP | 23.80516434 |  | RAB6A | 9.425477982 |
| MAP3K5 | 23.78909302 |  | DRD2 | 9.379380226 |
| SREBF1 | 23.3740654 |  | CASP7 | 9.365984917 |
| BCL2 | 23.12741661 |  | CCDC115 | 9.359241486 |
| PDIA4 | 23.11902237 |  | RNF5 | 9.354228973 |
| PPP1R15A | 22.87788773 |  | HIF1A | 9.339670181 |
| AMFR | 22.64559555 |  | HSD11B1 | 9.328950882 |
| TXNDC12 | 22.47172356 |  | MAP1LC3A | 9.28427887 |
| PSEN1 | 22.31509972 |  | HFE | 9.273126602 |
| DERL3 | 22.2395401 |  | PKD2 | 9.25092411 |
| STIM1 | 22.18220329 |  | OXT | 9.249069214 |
| ITPR1 | 22.16987419 |  | SHH | 9.24647522 |
| BCAP31 | 22.14373016 |  | UBQLN4 | 9.244839668 |
| HMOX1 | 22.09991455 |  | BRCA1 | 9.243590355 |
| SEC23A | 21.86993217 |  | GSTM1 | 9.230505943 |
| CASQ1 | 21.82103348 |  | F2 | 9.223731995 |
| MAN1B1 | 21.76463127 |  | RAC1 | 9.223232269 |
| MAPK14 | 21.64180756 |  | PPP1R15B | 9.222990036 |
| CASP3 | 21.38104248 |  | CYB5R3 | 9.216960907 |
| TNF | 21.37919617 |  | AGR2 | 9.213321686 |
| BAX | 21.26783752 |  | BOK | 9.201172829 |
| CALM1 | 21.00442696 |  | IL1A | 9.20032692 |
| TXNDC5 | 20.98152542 |  | DNAJB1 | 9.166652679 |
| DNAJB9 | 20.74643135 |  | ATL2 | 9.154561996 |
| CASP4 | 20.69721413 |  | COL7A1 | 9.145382881 |
| MBTPS2 | 20.52008057 |  | JPH4 | 9.140140533 |
| SAR1B | 20.50395775 |  | IFNG | 9.130059242 |
| H6PD | 20.20552635 |  | GPX1 | 9.120587349 |
| ATF6B | 20.05171776 |  | EEF1A1 | 9.111258507 |
|  |  |  |  |  |
| **Table S1. The 764 ER stress-related genes.** | | | | |
| Gene Symbol | Relevance score |  | Gene Symbol | Relevance score |
| CAT | 19.75525284 |  | AKAP9 | 9.105019569 |
| CREB3 | 19.49764633 |  | PMM2 | 9.1045084 |
| HSPA1A | 19.45793343 |  | LONP1 | 9.103369713 |
| CLU | 19.41539764 |  | CNR1 | 9.073740005 |
| MAPK1 | 19.387043 |  | SSR1 | 9.059407234 |
| STIP1 | 19.30893517 |  | TMX1 | 9.052736282 |
| SEL1L | 19.29961395 |  | SVIP | 9.045211792 |
| INS | 19.19307327 |  | IGF1 | 9.030948639 |
| DNAJB11 | 19.13234138 |  | JPH3 | 9.015399933 |
| PRNP | 18.93014526 |  | MAP2K4 | 9.005214691 |
| CFTR | 18.82065582 |  | SEC23IP | 8.998908997 |
| SLC6A4 | 18.67801285 |  | SCD | 8.990037918 |
| CISD2 | 18.67006874 |  | TRIB3 | 8.989543915 |
| G3BP1 | 18.62181091 |  | LDLR | 8.980133057 |
| OXSR1 | 18.11045456 |  | DHCR7 | 8.978527069 |
| CRH | 18.03106117 |  | ARL6IP5 | 8.967710495 |
| HSPA8 | 18.02435684 |  | PLEKHF2 | 8.94708252 |
| RTN4 | 17.94885635 |  | DMD | 8.945922852 |
| EPM2A | 17.70963097 |  | TMEM214 | 8.944759369 |
| MANF | 17.5620079 |  | SCAPER | 8.94389534 |
| PARK7 | 17.52607346 |  | SDHB | 8.938882828 |
| SEC61A1 | 17.34532547 |  | KDR | 8.865655899 |
| HSP90AA1 | 17.33255959 |  | PRDX2 | 8.86224556 |
| HSPA4 | 17.16532516 |  | ALG14 | 8.856385231 |
| IL6 | 17.0888443 |  | MR1 | 8.848080635 |
| RPN1 | 17.06507683 |  | MAP1LC3B | 8.831676483 |
| CREB3L1 | 16.93793678 |  | ATXN2 | 8.824477196 |
| FOS | 16.91001129 |  | PRDX1 | 8.81820488 |
| MIA2 | 16.67001724 |  | USO1 | 8.810408592 |
| SCN5A | 16.65118408 |  | EPO | 8.808393478 |
| BDNF | 16.54558945 |  | MUC1 | 8.805782318 |
| MAPK10 | 16.53118706 |  | PIGN | 8.801749229 |
| IL1B | 16.46382141 |  | SMPD4 | 8.798916817 |
| CYCS | 16.41096497 |  | UNC93B1 | 8.781152725 |
| SEC13 | 16.26172829 |  | STAU1 | 8.775942802 |
| ATP13A1 | 16.22333527 |  | FICD | 8.775619507 |
| SIRT1 | 16.12857819 |  | NPLOC4 | 8.762158394 |
| HSF1 | 15.99841404 |  | CAPN2 | 8.760388374 |
| DDRGK1 | 15.94338036 |  | NPC1 | 8.754130363 |
| EIF2AK2 | 15.89885426 |  | IL10 | 8.751125336 |
| PKP2 | 15.86741829 |  | CHAT | 8.736629486 |
|  |  |  |  |  |
| **Table S1. The 764 ER stress-related genes.** | | | | |
| Gene Symbol | Relevance score |  | Gene Symbol | Relevance score |
| SERPINA1 | 15.84617424 |  | MGST1 | 8.733103752 |
| SQSTM1 | 15.83697701 |  | COPA | 8.718706131 |
| VAPB | 15.80088806 |  | RNF186 | 8.716872215 |
| SEC31A | 15.78182983 |  | ORMDL3 | 8.698834419 |
| NOS3 | 15.4781456 |  | RNFT1 | 8.685599327 |
| CKAP4 | 15.32040882 |  | UBA52 | 8.681927681 |
| PDIA2 | 15.30674362 |  | CLN6 | 8.663898468 |
| ALB | 15.30348301 |  | SDF2L1 | 8.660752296 |
| GSR | 15.27534676 |  | OSBPL3 | 8.637096405 |
| CASP8 | 15.2198925 |  | GLA | 8.621865273 |
| MTOR | 15.19526386 |  | POR | 8.606319427 |
| SNCA | 15.11402702 |  | TMTC3 | 8.585639954 |
| HTT | 15.07762623 |  | SHISA5 | 8.57237339 |
| SEC24A | 15.01982307 |  | MICB | 8.572134018 |
| EIF2AK1 | 15.00073719 |  | NCK1 | 8.570668221 |
| EDEM1 | 14.95374393 |  | SERPINA3 | 8.568506241 |
| LMAN2 | 14.91388035 |  | GABARAP | 8.552855492 |
| SEC24B | 14.90847588 |  | GABARAPL1 | 8.548311234 |
| LRRK2 | 14.8592453 |  | UBA5 | 8.54818821 |
| PTPN1 | 14.84337711 |  | AIFM1 | 8.539351463 |
| INSIG1 | 14.79294395 |  | UGT1A1 | 8.528625488 |
| G3BP2 | 14.64332867 |  | GSTP1 | 8.524513245 |
| HSPB1 | 14.63860035 |  | PRKCSH | 8.516540527 |
| SOD2 | 14.59969521 |  | HM13 | 8.515169144 |
| AKT1 | 14.51945972 |  | HTR1A | 8.471476555 |
| DDOST | 14.51134682 |  | GRIN1 | 8.468221664 |
| SREBF2 | 14.45053196 |  | SCP2 | 8.461078644 |
| PSEN2 | 14.44229889 |  | TMCO1 | 8.443325043 |
| UBC | 14.44049644 |  | MDM2 | 8.443015099 |
| TRIP11 | 14.40368462 |  | NUPR1 | 8.427120209 |
| KCNH2 | 14.37622547 |  | UBE2D3 | 8.426844597 |
| ERLIN2 | 14.31218433 |  | CIB1 | 8.426324844 |
| SURF4 | 14.28301525 |  | HSPA6 | 8.422025681 |
| SIGMAR1 | 14.25002289 |  | ACTC1 | 8.406618118 |
| PLN | 14.23273182 |  | AKAP6 | 8.397034645 |
| CASP9 | 14.19370079 |  | TMX2 | 8.39572525 |
| RTN3 | 14.18481255 |  | CAMK2G | 8.383234978 |
| CREB3L2 | 14.16368866 |  | AGR3 | 8.377369881 |
| SSR4 | 14.08432865 |  | NR3C2 | 8.371743202 |
| NR3C1 | 14.08427429 |  | HMGB1 | 8.368024826 |
| UGGT1 | 14.07728195 |  | FN1 | 8.334354401 |
|  |  |  |  |  |
| **Table S1. The 764 ER stress-related genes.** | | | | |
| Gene Symbol | Relevance score |  | Gene Symbol | Relevance score |
| ANK2 | 14.03935051 |  | SNTA1 | 8.328302383 |
| SCAP | 14.00427055 |  | INSR | 8.325403214 |
| APOE | 13.98430634 |  | UBB | 8.325325966 |
| JUN | 13.97171307 |  | PTPN11 | 8.323208809 |
| ERGIC3 | 13.93023491 |  | CASP2 | 8.322829247 |
| BAK1 | 13.88363075 |  | ZC3H12A | 8.286047935 |
| DDX3X | 13.86972046 |  | CALHM1 | 8.279062271 |
| CALM3 | 13.85057259 |  | TYR | 8.270768166 |
| CREB3L3 | 13.83543205 |  | JPH1 | 8.261110306 |
| SEC62 | 13.8244009 |  | LMAN1L | 8.252773285 |
| CD4 | 13.79603577 |  | PTGS1 | 8.247514725 |
| ESR1 | 13.78534698 |  | CDK1 | 8.24095726 |
| MAPK9 | 13.78474998 |  | DAD1 | 8.236251831 |
| VEGFA | 13.78281784 |  | BET1 | 8.23254776 |
| TXN | 13.77262115 |  | CYP2D6 | 8.214920998 |
| KCNQ1 | 13.76457596 |  | ATXN3 | 8.214710236 |
| MPO | 13.67026043 |  | FKRP | 8.212198257 |
| MIA3 | 13.66897964 |  | SDHA | 8.207414627 |
| HMGCR | 13.63681602 |  | NAGLU | 8.194807053 |
| PDIA6 | 13.62106419 |  | PRDX6 | 8.194507599 |
| UBE2J1 | 13.59563732 |  | TLR9 | 8.191334724 |
| ADIPOQ | 13.57740593 |  | GET4 | 8.18813324 |
| TARDBP | 13.46494675 |  | TMEM43 | 8.184482574 |
| APOB | 13.38753891 |  | SSR3 | 8.184141159 |
| SAR1A | 13.34193707 |  | LPCAT3 | 8.179553986 |
| PARP1 | 13.33908272 |  | UBL4A | 8.166387558 |
| GJB2 | 13.31557083 |  | ATR | 8.161050797 |
| NHLRC1 | 13.31452465 |  | FBXO6 | 8.159557343 |
| LMNA | 13.29217052 |  | CNIH4 | 8.155877113 |
| SEC61B | 13.27563381 |  | UGT1A6 | 8.143083572 |
| PTGS2 | 13.26452351 |  | DNAJA1 | 8.140981674 |
| MOGS | 13.26423264 |  | CDKN3 | 8.117628098 |
| SEC63 | 13.21246243 |  | ICAM1 | 8.115354538 |
| SEC24C | 13.17729759 |  | YOD1 | 8.11423111 |
| POMC | 13.10021973 |  | UBE2K | 8.110961914 |
| ARL6IP1 | 13.08050537 |  | GABARAPL2 | 8.110215187 |
| EIF2AK4 | 13.04168415 |  | SCAMP5 | 8.080179214 |
| ITPR2 | 13.02485657 |  | CREBBP | 8.076352119 |
| TRAF2 | 13.01789951 |  | LBR | 8.062439919 |
| STUB1 | 12.99404144 |  | CLN8 | 8.053369522 |
| STIM2 | 12.99157715 |  | ZFAND2B | 8.042520523 |
|  |  |  |  |  |
| **Table S1. The 764 ER stress-related genes.** | | | | |
| Gene Symbol | Relevance score |  | Gene Symbol | Relevance score |
| TOR1A | 12.98473167 |  | JKAMP | 8.040967941 |
| ASPH | 12.98285866 |  | NR1H2 | 8.034925461 |
| CAV1 | 12.97164631 |  | UFM1 | 8.034923553 |
| ERLIN1 | 12.96363354 |  | HMGCLL1 | 8.026309967 |
| GJA1 | 12.94805908 |  | CALR3 | 8.009028435 |
| KEAP1 | 12.93227577 |  | SEC11A | 7.983335018 |
| RPN2 | 12.92322731 |  | CARD14 | 7.982457638 |
| EBP | 12.91673851 |  | SLC39A14 | 7.977982044 |
| ATF3 | 12.8856163 |  | GRIN2A | 7.964386463 |
| MAPT | 12.88403702 |  | RINT1 | 7.96286869 |
| UBQLN1 | 12.88007736 |  | UBE2D2 | 7.962516308 |
| TGFB1 | 12.84081268 |  | PDZD8 | 7.951392174 |
| HSPA1B | 12.83945942 |  | PRKCA | 7.951264381 |
| CALM2 | 12.8375206 |  | IKBKG | 7.949530125 |
| UGGT2 | 12.81234837 |  | G6PC3 | 7.948877811 |
| TMED4 | 12.81095028 |  | SGPP1 | 7.947796822 |
| CRHR1 | 12.79332447 |  | TGM2 | 7.931829453 |
| ORAI1 | 12.7824173 |  | MAPKAP1 | 7.930656433 |
| INPP5K | 12.76670074 |  | DBH | 7.92550087 |
| TAPBP | 12.72055626 |  | GPX7 | 7.924183369 |
| CLN3 | 12.70144653 |  | NPY | 7.914775848 |
| CCL2 | 12.68483734 |  | CDK5RAP3 | 7.910202026 |
| VWF | 12.67286777 |  | SHC1 | 7.887420654 |
| RNF185 | 12.66768074 |  | NDRG1 | 7.885954857 |
| P4HTM | 12.6570797 |  | APOA1 | 7.882487297 |
| TAP1 | 12.62128258 |  | NSFL1C | 7.878029346 |
| MBTPS1 | 12.61299229 |  | SCARA3 | 7.876515388 |
| BNIP1 | 12.60292149 |  | MSRB3 | 7.873408318 |
| DSP | 12.55097198 |  | EEF2 | 7.872938633 |
| PREB | 12.53696537 |  | KDSR | 7.872220993 |
| INSIG2 | 12.53026581 |  | FAS | 7.866446495 |
| CACNA1C | 12.46647739 |  | ATP1A3 | 7.859384537 |
| EDEM2 | 12.44312477 |  | REEP4 | 7.858773232 |
| PPIB | 12.419034 |  | SGTA | 7.8577075 |
| HSPA9 | 12.41757679 |  | ALG11 | 7.856297493 |
| SESN2 | 12.40770149 |  | GBA2 | 7.846226692 |
| NOTCH3 | 12.40566635 |  | PITPNB | 7.820402145 |
| BSCL2 | 12.37554646 |  | TERT | 7.813932896 |
| SEC24D | 12.35560036 |  | FKBP1B | 7.812069893 |
| CRP | 12.33160496 |  | RAB2A | 7.811389446 |
| DPM1 | 12.32529259 |  | ADRB2 | 7.796595097 |
|  |  |  |  |  |
| **Table S1. The 764 ER stress-related genes.** | | | | |
| Gene Symbol | Relevance score |  | Gene Symbol | Relevance score |
| JPH2 | 12.29231644 |  | TNFRSF1A | 7.785863876 |
| NOX4 | 12.28901672 |  | PABPC1 | 7.776647091 |
| VIM | 12.28285122 |  | MET | 7.766469002 |
| MYH7 | 12.20012474 |  | MAP2K1 | 7.76312685 |
| ALG2 | 12.12246323 |  | BCL2L10 | 7.745137691 |
| ABL1 | 12.10653019 |  | DES | 7.743538857 |
| EDEM3 | 12.08042336 |  | FUS | 7.737304688 |
| TMED2 | 12.06261444 |  | STK4 | 7.727864265 |
| RPS27A | 12.06103516 |  | ANXA5 | 7.723806381 |
| RSAD2 | 12.0469141 |  | AGER | 7.700470924 |
| SLN | 11.95949078 |  | PTPN2 | 7.693512917 |
| CAV3 | 11.94992447 |  | PIK3CA | 7.686023712 |
| CRYAB | 11.93026924 |  | CDK5 | 7.680241585 |
| ESYT1 | 11.90175533 |  | GOLGA2 | 7.679141998 |
| SGK1 | 11.87173557 |  | SRP54 | 7.670682907 |
| RRBP1 | 11.84344769 |  | MYC | 7.666689873 |
| BCL2L1 | 11.84061623 |  | DGAT1 | 7.663871765 |
| DNAH8 | 11.74106026 |  | SUMF2 | 7.659487724 |
| EIF4G1 | 11.72704887 |  | ZFYVE27 | 7.655930042 |
| EGF | 11.7078352 |  | ATP6AP1 | 7.64435482 |
| STT3A | 11.65719318 |  | PKD1 | 7.643932343 |
| HLA-B | 11.65643501 |  | COL2A1 | 7.634986877 |
| MYOC | 11.65222073 |  | MFN2 | 7.620899677 |
| PRKCD | 11.61937141 |  | TECR | 7.617012501 |
| BECN1 | 11.61693478 |  | PTGIS | 7.614570618 |
| MAP2K7 | 11.61003399 |  | HLA-DRB1 | 7.614323616 |
| TMBIM6 | 11.59458447 |  | PRL | 7.602875233 |
| YIPF5 | 11.56074238 |  | STAT3 | 7.602003574 |
| UBE2G2 | 11.55295277 |  | RCN1 | 7.585334778 |
| EGFR | 11.53953266 |  | SOAT2 | 7.581136703 |
| FKBP14 | 11.53550053 |  | NOL3 | 7.578105927 |
| SERPINH1 | 11.53119946 |  | GORASP2 | 7.577799797 |
| ATM | 11.49878216 |  | CCND1 | 7.569014072 |
| RAB1B | 11.49517155 |  | CTSD | 7.568810463 |
| PON1 | 11.45652676 |  | S100A1 | 7.568521023 |
| GBF1 | 11.42034435 |  | TFRC | 7.567745686 |
| CANT1 | 11.41843891 |  | SACM1L | 7.565867901 |
| PRKAA1 | 11.4018383 |  | YKT6 | 7.564786911 |
| ATL1 | 11.3884964 |  | MX1 | 7.562233925 |
| COMT | 11.38242149 |  | PRDX3 | 7.548365116 |
| TLR4 | 11.37531662 |  | HSPA2 | 7.547213554 |
|  |  |  |  |  |
| **Table S1. The 764 ER stress-related genes.** | | | | |
| Gene Symbol | Relevance score |  | Gene Symbol | Relevance score |
| FOXO3 | 11.36980247 |  | PACS2 | 7.544091225 |
| NFKB1 | 11.3628521 |  | RNF183 | 7.537138939 |
| HTRA2 | 11.34379387 |  | STARD3NL | 7.536044121 |
| RNF139 | 11.30942726 |  | HSPA1L | 7.535622597 |
| GAPDH | 11.29572868 |  | FGFR3 | 7.532170296 |
| ERGIC2 | 11.28324699 |  | SET | 7.502313137 |
| XDH | 11.26708603 |  | HTR2A | 7.495811939 |
| FMR1 | 11.21473503 |  | MICA | 7.489530563 |
| VAPA | 11.19029427 |  | REEP1 | 7.477824211 |
| PRDX4 | 11.15846634 |  | TF | 7.474067211 |
| HSD17B10 | 11.14091778 |  | FURIN | 7.459943771 |
| TAP2 | 11.13318634 |  | PIK3CG | 7.4523592 |
| SLC8A1 | 11.12768555 |  | TH | 7.443890572 |
| RAB1A | 11.12703896 |  | DSG2 | 7.439429283 |
| RTN1 | 11.12240982 |  | TTN | 7.427312851 |
| HSP90AB1 | 11.11607361 |  | MPPE1 | 7.426245689 |
| ALG1 | 11.09031105 |  | MMP2 | 7.413210869 |
| CYBA | 11.07456017 |  | TMED7 | 7.40729475 |
| KCNE1 | 11.07266808 |  | MMP9 | 7.405224323 |
| PINK1 | 11.05424404 |  | DLD | 7.402472019 |
| BACE1 | 11.05321312 |  | PPM1L | 7.397455215 |
| SLC37A4 | 11.05242634 |  | PITPNM1 | 7.380183697 |
| TOR1B | 11.04020023 |  | MAOB | 7.376048565 |
| NLRP3 | 11.02379036 |  | PDHA1 | 7.373928547 |
| TRIM13 | 11.01077461 |  | F8 | 7.368185997 |
| UBXN8 | 10.99821758 |  | EIF4E | 7.362925529 |
| SEC61G | 10.98072243 |  | NOS1 | 7.359558105 |
| TMEM33 | 10.96891022 |  | FKBP4 | 7.349718571 |
| CASP12 | 10.96401978 |  | CP | 7.347126961 |
| GBA | 10.96096897 |  | RELA | 7.347102642 |
| FOXO1 | 10.95375538 |  | MCFD2 | 7.345672607 |
| UBE2J2 | 10.94944859 |  | VDAC1 | 7.344763756 |
| SOAT1 | 10.93409443 |  | PML | 7.340950012 |
| MAPK3 | 10.890378 |  | F9 | 7.328705788 |
| TEX264 | 10.88773632 |  | CLGN | 7.326984406 |
| OSBPL8 | 10.85875893 |  | GRAMD1A | 7.321730137 |
| COPB1 | 10.84566307 |  | SYT2 | 7.317959785 |
| HERPUD2 | 10.82045269 |  | SLC35B1 | 7.314110756 |
| TECRL | 10.81822205 |  | CUL1 | 7.310106277 |
| CEBPB | 10.80352974 |  | FBN1 | 7.298187256 |
| TRAM1 | 10.77105331 |  | EIF2B5 | 7.298056126 |
|  |  |  |  |  |
| **Table S1. The 764 ER stress-related genes.** | | | | |
| Gene Symbol | Relevance score |  | Gene Symbol | Relevance score |
| NOS2 | 10.7501955 |  | UBAC2 | 7.288791656 |
| PDCD6 | 10.74252892 |  | CYP1B1 | 7.282823563 |
| PRKAA2 | 10.73916531 |  | LEP | 7.278378487 |
| STX17 | 10.68334389 |  | BRCA2 | 7.27555418 |
| KCNJ5 | 10.6713028 |  | CRYAA | 7.267798424 |
| GSK3B | 10.66794395 |  | MOSPD2 | 7.261269093 |
| RAB10 | 10.66620922 |  | SRPRB | 7.250794411 |
| BBC3 | 10.63701057 |  | TRPM4 | 7.243917465 |
| DNAJB12 | 10.6347208 |  | GOSR2 | 7.241415501 |
| TNFRSF10B | 10.61641693 |  | KRAS | 7.232588768 |
| CCDC47 | 10.59065056 |  | BTRC | 7.216765404 |
| NQO1 | 10.58208275 |  | CD74 | 7.21405983 |
| C9orf72 | 10.58016205 |  | GRAMD1B | 7.212460518 |
| VHL | 10.55680656 |  | ELAVL1 | 7.208126068 |
| CTNNB1 | 10.54771042 |  | USH2A | 7.207623482 |
| SEC23B | 10.53989887 |  | TMED1 | 7.196993828 |
| DHCR24 | 10.53023911 |  | TMEM117 | 7.194215775 |
| TMEM208 | 10.50498009 |  | CRHR2 | 7.180266857 |
| FAF2 | 10.49123764 |  | HSD17B12 | 7.171414375 |
| CCDC88B | 10.46766949 |  | RHBDD1 | 7.161537647 |
| SPAST | 10.45716476 |  | DHDDS | 7.161227703 |
| ITPR3 | 10.43586254 |  | CLCC1 | 7.156112671 |
| AHCYL1 | 10.42249012 |  | DSPP | 7.14531374 |
| AUP1 | 10.41230965 |  | CDC42 | 7.141435623 |
| DNAJB2 | 10.3822794 |  | GRIN2B | 7.13926506 |
| IER3IP1 | 10.37684536 |  | UBE2D1 | 7.120244026 |
| CYP2E1 | 10.36587143 |  | HLA-DRA | 7.118727684 |
| STT3B | 10.35967255 |  | GHRL | 7.103425503 |
| TXNIP | 10.34432602 |  | TRPA1 | 7.102540016 |
| AQP11 | 10.31196785 |  | TRPM2 | 7.099803925 |
| GPR37 | 10.30900764 |  | GATA1 | 7.096940041 |
| TG | 10.29136086 |  | ABCD4 | 7.094830036 |
| SCN4A | 10.27717686 |  | BAG1 | 7.0914464 |
| FOXRED2 | 10.26811314 |  | SERPINE1 | 7.08880043 |
| TMED9 | 10.24859047 |  | KPNB1 | 7.086412907 |
| PIK3R1 | 10.21653748 |  | ACTG1 | 7.086343765 |
| PCSK9 | 10.20112705 |  | DSC2 | 7.083759308 |
| PPARG | 10.19586182 |  | C1R | 7.081218719 |
| EDN1 | 10.19405174 |  | TTR | 7.075907707 |
| MTTP | 10.19033146 |  | SRP72 | 7.071773052 |
| CREB1 | 10.15229607 |  | PRDX5 | 7.070888519 |
|  |  |  |  |  |
| **Table S1. The 764 ER stress-related genes.** | | | | |
| Gene Symbol | Relevance score |  | Gene Symbol | Relevance score |
| CYP1A2 | 10.13426208 |  | MYDGF | 7.064078808 |
| RCN2 | 10.12371254 |  | USP19 | 7.060030937 |
| TUSC3 | 10.11118984 |  | CAPN1 | 7.054354668 |
| MAPKAPK2 | 10.09593582 |  | PLA2G6 | 7.053322792 |
| TFG | 10.09069252 |  | ZFYVE1 | 7.042645931 |
| DPAGT1 | 10.08526802 |  | SCFD1 | 7.040589333 |
| G6PD | 10.07720089 |  | PLOD3 | 7.033716679 |
| ESYT2 | 10.07236767 |  | CYB5R4 | 7.017508507 |
| FKBP5 | 10.07215881 |  | NLRP1 | 7.017127991 |
| UBXN4 | 10.06620026 |  | KNG1 | 7.014955044 |
| STARD3 | 10.05383873 |  | GFAP | 7.008621216 |
| KTN1 | 10.03781796 |  | DST | 7.003973007 |
