# Supplementary material for: Development and validation of a prognosis prediction model based on 18 endoplasmic reticulum stress-related genes for patients with lung adenocarcinoma
Source: Front Oncol. 2022 Aug 30;12:902353. doi: 10.3389/fonc.2022.902353 (PMC9469654; doi:10.3389/fonc.2022.902353)
Supplement: Supplementary file 2 [file DataSheet_1.pdf]

Forestplot

1. Import rawdata

Univariate cox regression analyses were performed to calculate the HR and P value of ERSS in different subgroup of patients. Next, the results should be recorded in excel as following form:

	A	B	C	D	E	F	G
1	Subgroups	Number of patients	HR (95%CI)				P.value
2	ALL	504	3.99 (2.95, 5.39)	3.99	2.95	5.39	<0.001
3	Age/years						
4	≥65	274	4.65 (3.05, 7.08)	4.65	3.05	7.08	<0.001
5	<65	220	3.95 (2.48, 6.29)	3.95	2.48	6.29	<0.001
6	Unknown	10	1.45 (0.19, 11.09)	1.45	0.19	11.09	0.718
7	Gender						
8	Male	233	4.47 (2.90, 6.90)	4.47	2.9	6.9	<0.001
9	Femal	271	3.51 (2.30, 5.35)	3.51	2.3	5.35	<0.001
10	Smoking_history						
11	Yes	417	3.85 (2.76, 5.36)	3.85	2.76	5.36	<0.001
12	No	73	4.67 (1.95, 11.20)	4.67	1.95	11.2	0.001
13	Unknown	14	4.48 (0.96, 20.86)	4.48	0.96	20.86	0.056
14	Stage						
15	I	271	3.12 (1.85, 5.27)	3.12	1.85	5.27	<0.001
16	II	120	3.27 (1.85, 5.76)	3.27	1.85	5.76	<0.001
17	III	80	4.71 (2.45, 9.06)	4.71	2.45	9.06	<0.001
18	IV	25	5.24 (1.67, 16.45)	5.24	1.67	16.45	0.005
19	Unknown	8	3.99 (2.95, 5.39)	3.99	2.95	5.39	<0.001

```
library(openxlsx)
forest <- read.xlsx("rawdata/Sub_anlyasis_unicox.xlsx",colNames = F) #Import the data
```

2. Clean the data

```
subgps <- c(4:6,8,9,11:13,15:19) #Specify the subgroup to indent, where the number in the vector represents the number of rows in the subgroup
forest$X1[subgps] <- paste("    ",forest$X1[subgps]) #Add two spaces before the subgroup
labeltext <- as.matrix(forest[,c(1:3,7)])
```

3. Make the forest plot

```
library(forestplot)
forestplot(labeltext = labeltext,
           txt_gp = fpTxtGp(cex = 0.7),
           mean=forest$X4,
           lower=forest$X5,
           upper=forest$X6,
           is.summary=c(T,T,
                        T,F,F,F,
                        T,F,F,
                        T,F,F,F,
                        T,F,F,F,F,F),
           zero=1,
           boxsize=0.3,
           lineheight = unit(8,'mm'),
           colgap = unit(4,'mm'),
           lwd.zero = 2,
```

```
            lwd.ci = 2,
            col=fpColors(box='#458B00',summary="#8B008B",lines = 'black',zero
='#7AC5CD'),
            xlab="The estimates",
            lwd.xaxis=2,
            lty.ci = "solid",
            graph.pos = 4,
            graphwidth=unit(40,'mm'))
```
